# Accomplishments of “Old-Fashioned” Electron Microscopy in the Period of Dominance of Immunofluorescent Methods

**DOI:** 10.3390/ijms27062803

**Published:** 2026-03-19

**Authors:** Yury M. Morozov, Pasko Rakic

**Affiliations:** Department of Neuroscience, Kavli Institute for Neuroscience, Yale University School of Medicine, 333 Cedar Street, SHM, C-316, New Haven, CT 06520-8001, USA

**Keywords:** cannabinoids, biomarkers, specificity of the antibodies, brain development, cellular necrosis, mitochondria, Golgi apparatus, centrosome anchorage

## Abstract

The goal of this review is to bring to the attention of the scientific community the opportunities of transmission electron microscopy for analyses of biological subjects and resolving complicated cases of data interpretation. Although procedures for electron microscopy are in general more elaborate (particularly for simultaneous immunolabeling of multiple antigens) compared to fluorescent microscopy, they can help view cellular morpho-functional features undetectable using other methods. In this review, we consider several unexpected and serendipitous discoveries made in our laboratory and fulfilled using unique opportunities provided by electron microscopy of ultrathin sections. We are deliberating the following topics: interpretation of unusual results of immunolabeling; a novel method for in situ identification of cells undergoing mitochondrial disorder and necrosis-like death; the sequence of organelles’ reorganization in dying cells; simultaneous rupture of nuclear and plasma membranes in migrating neurons; and the role of cytoskeleton in lateral expansion of the cerebral cortex.

## 1. Introduction

Nowadays, if a general biologist is asked “What is the main benefit of transmission electron microscopy?”, they will likely answer: “It has very high magnification and resolution”. Such an answer, while correct, does not capture the entire picture. Indeed, the focus on high resolution elicits another question that does not have a proper answer: “For what purpose do we need extremely high resolution if that which fluorescent microscopy provides is sufficient?” The main benefit of electron microscopy is not its unbeatable magnification and resolution but the freedom to analyze all the cells and organelles regardless of immunolabeling or any selective staining procedure. This provides an important advantage in comparison with most types of light microscopy, including modern confocal, multiphoton, and super-resolution methods that visualize only stained cells or molecules. As an advantage of immunofluorescent microscopy, we must highlight the relative simplicity to perform multiple antigen labeling (triple and more), whereas double labeling for transmission electron microscopy is much more elaborate. Nevertheless, electron microscopy combined with immunolabeling provides not only an opportunity to analyze the labeled cells and organelles but also to gain context from the surrounding structures. Specifically, this can help with the correct interpretation of immunolabeling or to identify misleading artifacts. A combination of electron microscopy with light microscopy for analysis of the same sample, so-called correlative microscopy, can be extremely useful by harnessing the advantages of both methods. Light microscopy can analyze a large area of tissue, whereas electron microscopy lets the researcher investigate chosen cells or tissue segments in detail. Here we review unexpected and serendipitous discoveries made in our laboratory using unique opportunities provided by electron microscopy combined with immunolabeling, light microscopy and three-dimensional (3D) reconstruction from serial ultrathin sections. In particular, we consider the following aspects: (1) double selectivity of antibodies producing unusual immunolabeling of mitochondria that helps for in situ identification of cells undergoing mitochondrial disorder and necrosis-like cell death; (2) the sequence of morpho-functional reorganization of mitochondria and Golgi apparatus in anoxia-exposed mouse embryo brains; (3) a novel mechanism of cell pathology identified through the simultaneous rupture of nuclear and plasma membranes in neurons migrating through tightly packed brain tissue; and (4) the role of centrosomes—the main cytoskeleton organizing center—in prolonged lateral expansion of rhesus macaque’s cerebral cortex.

## 2. Interpretation of Anti-Cannabinoid Type 1 Receptor Sera Binding with Mitochondria

**Figure 1:**
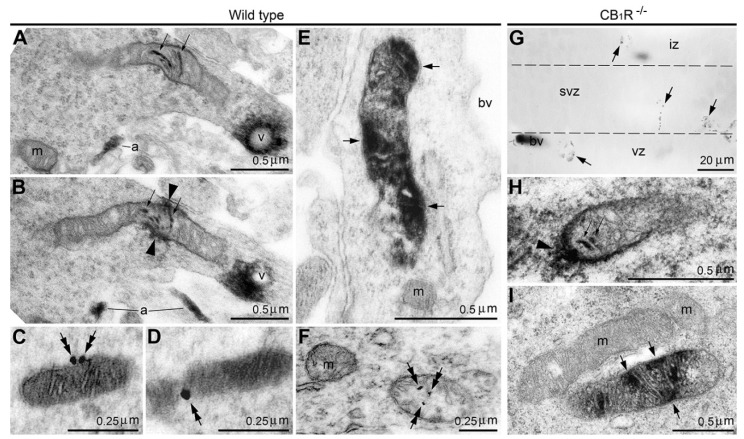
Two types of anti-CB_1_R immunolabeling of mitochondria in the immature cerebrum of wild-type (**A**–**F**) and CB_1_R^−/−^ (**G**–**I**) mice obtained using anti-CB_1_R serum. (**A**–**D**) Serial micrographs of type 1 mitochondria. (**A**,**B**) Notice nickel-intensified 3,3′-diaminobenzidine-4HCl (DAB-Ni) immunoprecipitation in the cristae (small arrows) and around the mitochondrion (arrowheads). Known patterns of anti-CB_1_R immunolabeling are also visible, namely, in the neural processes—putative axons (a) and on the outer surface of intracellular vesicles (v). (**C**,**D**) Immunogold–silver particles (double arrows) on the outer surface of the mitochondrion seen in serial sections. (**E**,**F**) DAB-Ni immunoprecipitation (arrows in **E**) and immunogold–silver particles (double arrows in **F**) label the matrix of type 2 mitochondria, whereas cristae remain immunonegative. (**G**–**I**) Anti-CB_1_R immunolabeling in the CB_1_R^−/−^ mouse embryo cerebrum. (**G**) Light micrograph of the neocortex demonstrates numerous immunopositive particles (arrows) in the neuropil and blood vessels (bv), whereas CB_1_R-expressing axons and cell bodies are absent. (**H**,**I**) Electron micrographs show that immunopositive particles seen in (**G**) represent mitochondria of both types identical to the labeling detected in wild-type animals. Notice stained cristae (small arrows) and staining outside the mitochondrion (arrowhead in **H**) and matrix staining (arrows in **I**). The borders between ventricular (vz), subventricular (svz) and intermediate (iz) zones of the embryonic cerebrum are demarked with dashed lines. Abbreviation: m, immunonegative mitochondria. Modified from [6]. (License 6183810481618).

The use of antibodies for immunohistochemical identification of proteins has resulted in striking advances in cellular and molecular research of complex biological systems. Nevertheless, there are several technical issues that need to be taken into consideration. In particular, the molecular construction of the antigen’s antibody-binding site—the epitope, may be conformational, i.e., composed of discontinuous sections of the amino acid sequence that are brought together through a secondary or tertiary protein structure. In such cases, precise analysis of the epitope is an extremely complicated and time-intensive procedure that is impractical to perform and has not been performed for thousands of commercially available sera currently used for scientific research [1,2,3,4,5]. As a result, immunolabeling of conformational epitopes may depend on the experimental conditions, making serological identification of proteins uncertain and prone to misinterpretation. For example, in our study of cannabinoid signaling in the mammalian brain, we detected binding of anti-cannabinoid type 1 receptor (CB_1_R) sera with mitochondria of two morpho-functional types, whereas many other mitochondria stay immunonegative (Figure 1A–F and [6]). One type of immunopositive mitochondria, designated as “type 1”, contained immunoreaction end products on the outer membrane and inside the cristae. The other type, designated “type 2”, showed immunolabeling in the mitochondrial matrix. (See next chapters for discussion of probable functionality of these mitochondrial types.) At first glance, this might be interpreted as evidence of this G-protein-coupled receptor’s functionality in a new location in the mitochondria [7,8]. Mitochondrial immunolabeling takes place in parallel with well-documented immunolabeling of CB_1_R in the axonal plasma membrane and intracellular vesicles in neuronal cell bodies confirming selectivity of the applied serum for detection of CB_1_R (reviewed in [9,10,11,12,13,14,15]). However, negative control showed equal labeling of mitochondria in wild-type and CB_1_R-knock-out (CB_1_R^−/−^) mice, demonstrating non-CB_1_R binding of the antibodies (Figure 1G–I). After thorough analysis, we identified that, in parallel with CB_1_R, the applied anti-CB_1_R serum also binds to a conformational epitope of the mitochondrial protein stomatin-like protein-2 (SLP2) [6,16]. In accordance, several analyzed agonists and antagonists of CB_1_R produced irregular effects on the respiratory activity of the isolated mitochondria, suggesting their direct action to the electron transport chain complexes or to the lipid components of the mitochondrial membranes rather than to the suspected cannabinoid receptor in the mitochondria [17]. Thus, the visible location and functionality of CB_1_R in mitochondria has an alternative explanation. Overall, extra caution should be taken when interpreting unusual types of immunolabeling. In such cases, a proper application of electron microscopy can help.

## 3. Anti-CB_1_R Serum Detects Disordered Mitochondria and Damaged Cells in Mouse Brain

In our light and electron microscopy analyses of CB_1_R expression in the developing and adult mouse brain, we often utilized a combination of two visualization methods: (i) a highly sensitive method of immunoperoxidase reaction with nickel-intensified 3,3′-diaminobenzidine-4HCl (DAB-Ni) as a chromogen; and (ii) pre-embedding an ultra-small gold immunolabeling procedure with silver amplification that is less sensitive but provides more precise antigen location. This enabled us to detect two patterns of anti-CB_1_R sera binding to mitochondria that likely indicate SLP2 rather than CB_1_R. The best results were obtained with polyclonal antibodies raised against the last 31 amino acids of the C-terminus (L31) of CB_1_R [6]. One population of the immunopositive mitochondria, designated as type 1, contained DAB-Ni immunoreaction end products on the outer membrane and inside the cristae (Figure 1A,B,H). The location of the antigen on the outer surface of the mitochondrial membrane, but not in the cristae, was confirmed using immunogold labeling (Figure 1C,D). The other type of immunopositive mitochondria, designated as type 2, contained the antigen in the matrix—also confirmed using immunogold labeling (Figure 1E,F,I). The specificity of these immunolabeling patterns is supported by our data showing that pre-absorption of the anti-CB_1_R sera with the fusion peptide (L31) abrogated binding of the antibodies [6].

**Figure 2:**
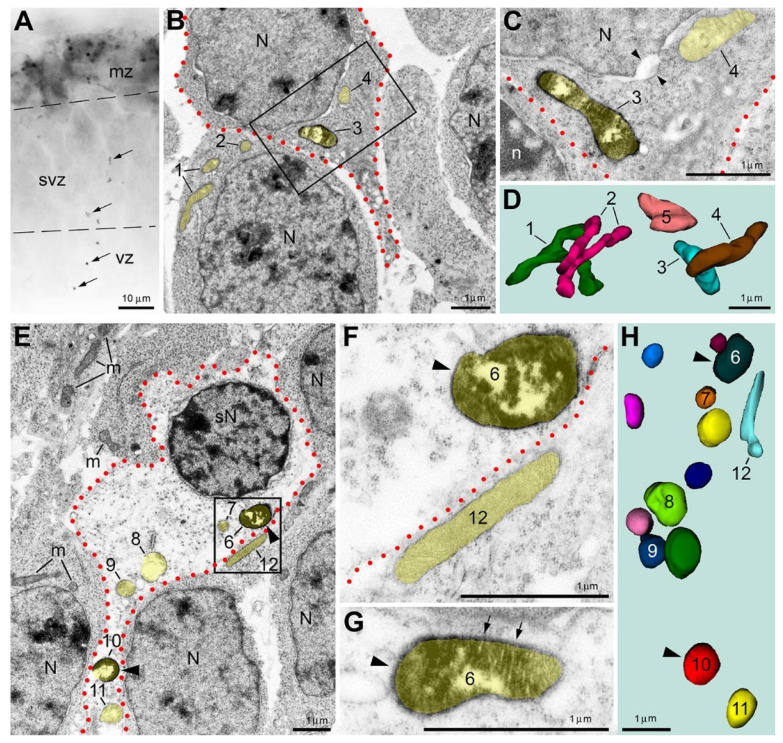
Immunopositive mitochondria-containing neurons detected with anti-CB_1_R-L31 serum show minor (**A**–**D**) or major ultrastructural pathology (**E**–**H**) in the mouse embryo cerebrum. (**A**) Light microscopy reveals robust CB_1_R immunolabeling in the marginal zone (mz), which is characteristic for developing interneurons, as well as a subset of immunopositive mitochondria (arrows) in the ventricular zone (vz) and subventricular zone (svz). (**B**–**D**) Electron microscopy with 3D reconstruction from the tissue segment displayed in (**A**) demonstrates an immunopositive mitochondria-containing neuron (outlined with red dotted line) with minor ultrastructural pathology, such as enlarged nuclear membrane (coupled arrowheads in **C**). Mitochondrial profiles are highlighted yellow and respectively numbered in the electron micrographs and corresponding 3D images. Both immunopositive (e.g., #3) and immunonegative mitochondria (e.g., #4 and #5) in this neuron are slightly swollen. Thin branched mitochondria #1 and #2 exemplify normal mitochondria of immunonegative neurons. The framed area in (**B**) is shown in (**C**) as a high-power micrograph taken from a serial section. (**E**–**H**) A neuron (outlined with red dotted line) with major ultrastructural pathology [such as spherical nucleus (sN) and near-translucent cytoplasm] contains multiple swollen, spherical mitochondria that could be either immunopositive [arrowheads, #6 (shown with magnified serial images in **F**,**G**) and #10] or immunonegative (#7–9 and #11). In contrast, the surrounding immunonegative neurons contain morphologically normal mitochondria (m; e.g., #12), ovoid nuclei (N) and normal cytoplasm characteristic of developing neurons. Modified from [16]. (License 6183801444721).

Systematic analysis of serial ultrathin sections paired with 3D reconstruction of cells and organelles considerably increases the scientific value of electron microscopy study of biological subjects [18,19]. For example, modern automatic systems for volume electron microscopy analysis (e.g., FIB-SEM—Focused Ion Beam Scanning Electron Microscopy) are very effective but require expensive specialized equipment. In our studies, we performed 3D reconstructions using universally applicable transmission electron microscopy methods and publicly available Reconstruct software version 1.1.0.0 [20,21,22], which do not require additional expenses. Application of 3D reconstruction enabled us to demonstrate that type 1 immunopositive mitochondria had, on average, the same length and diameter as immunonegative mitochondria [16]. In contrast, the type 2 mitochondria demonstrated a variety of ultrastructural pathologies, ranging from a slightly increased diameter to distinct swelling and lysis of the inner compartment (Figure 2). Notably, emergence of the type 2 (but not the type 1) mitochondria is linked to disorder of the cells. In the normal developing mouse brain, we detected sporadic spots of neuropil containing immunopositive type 2 mitochondria, while surrounding tissue was immunonegative (Figure 2A,B). Electron microscopy analysis revealed that some of the type 2 mitochondria-containing cells demonstrated minor ultrastructural pathology, namely, slightly swollen mitochondria (regardless of their immunopositive or negative status) as well as swollen nuclear membranes and endoplasmic reticulum cisterns (Figure 2C,D). Other cells showed severe ultrastructural pathology, nearing complete degradation of cytoplasm (Figure 2E–H). Regardless of the degree of damage in the type 2 mitochondria-containing cells, adjacent cells containing only immunonegative mitochondria demonstrated an ultrastructure characteristic of normal developing neurons. Thus, the observed phenotype of the type 2 mitochondria provides a new target for in situ diagnosis of cellular degeneration.

## 4. What CB_1_R-L31 Immunolabeling Reveals About Mitochondrial Function

Owing to its central role in energy supply and several other aspects of cellular physiology and regulated cell death, the mitochondria have been heavily studied (reviewed in [23,24,25,26]). This has led to many immunological and biochemical methods for assessing their functionality and dysregulation. However, most current methods are applicable only to cultured cells or require organelle fractionation, whereas there is a lack of histochemical approaches to detect disordered mitochondria in intact organs. Our findings on immunohistochemical detection of SLP2 may fill this void and offer new insights into morpho-functional alterations of mitochondria in situ. SLP2 is a constitutive mitochondrial protein that is incorporated in the inner mitochondrial membrane and plays a role in mitochondrial functionality interacting with phospholipids and prohibitins, or creating the hetero-oligomer complex with mitofusin 2 [27,28,29,30,31]. According to a recent publication, SLP2 is attached to the inner mitochondrial membrane facing the mitochondrial matrix. SLP2 plays a role in the anchoring and spatial organization of the protease complex composed of the rhomboid protease PARL and the *i*-AAA protease YME1L in the inner mitochondrial membrane. SLP2, together with the protease complex, regulates mitochondrial dynamics, quality control, and cell survival [32].

**Figure 3:**
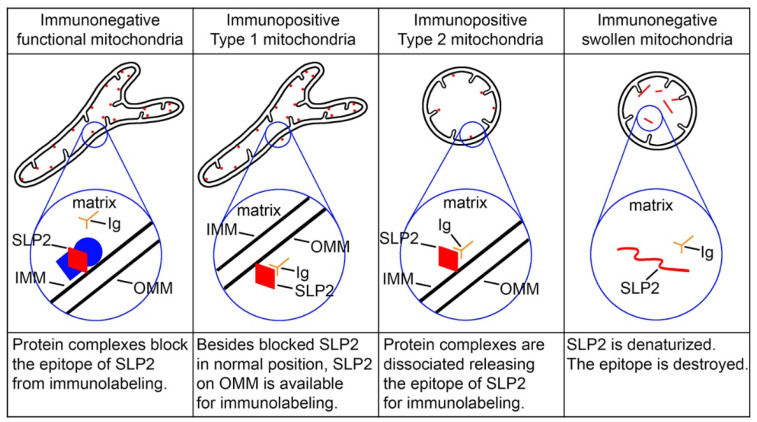
Hypothetical model of relationships between the mitochondrial morphology, molecular conformation of the SLP2 protein (red), and CB_1_R-L31 antibodies (Ig). Unidentified proteins that make a complex with SLP2 on the inner surface of the inner mitochondrial membrane (IMM) are depicted in blue. SLP2 deposited on the outer mitochondrial membrane (OMM), likely involved in the subsequent translocation into the mitochondrion, binds CB_1_R-L31 antibodies in the type 1 mitochondria. Dissociation of the protein complex makes SLP2 available for immunolabeling in the type 2 mitochondria, whereas further disorder of mitochondrion denatures SLP2. Modified from [33]. (License CC BY).

Because only one unique CB_1_R-immunopositive band was visible in our Western blot analysis of mitochondrial fractions [6], we hypothesized that SLP2 is detectable in both type 1 and type 2 mitochondria. Although SLP2 is a constitutive mitochondrial protein and likely expressed in all mitochondria, CB_1_R-L31 serum detected it in a relatively small number of mitochondria. We hypothesized that the previously demonstrated tight contacts between SLP2 and other molecules [27,28,29,32] block its epitope in the functional mitochondria from binding with CB_1_R-L31 antibodies as well as with currently available commercial SLP2 antibodies. This explains why most mitochondria with normal morphology are immunonegative (Figure 3). Release of SLP2 from tight contacts with other molecules frees it up for interaction with CB_1_R-L31 antibodies in type 1 and type 2 mitochondria. Type 1 mitochondria, which show the ultrastructure characteristic for normal functionality, contain immunolabeling end products on the outer membrane and inside the cristae, of which only the outer membrane location was confirmed through immunogold labeling (Figure 1A–D). Such an immunolabeling pattern likely indicates that new SLP2 molecules synthesized in cytoplasm are docked on the mitochondrion surface for subsequent transportation inside the organelle (Figure 3). DAB-Ni depositions may diffuse inside the crista through the pores or tiny perforations in the outer mitochondrial membrane. If so, DAB-Ni depositions in the crista represent an artifact and should be ignored. In general, type 1 SLP2-like immunopositive mitochondria do not differ from normal immunonegative mitochondria. In contrast, the anti-CB_1_R-L31 serum labels matrix of the type 2 mitochondria that are either moderately or dramatically swollen (Figure 2 and Figure 3). This corresponds with the modern view on the location of SLP2 on the inner surface of the inner mitochondrial membrane [32]. In such a way, anti-CB_1_R-L31 labeling may detect cells with early or advanced stages of pathology. Some mitochondria, although dramatically swollen, remain immunonegative—likely a result of SLP2’s denaturation destroying the epitope for CB_1_R-L31 antibodies (Figure 2 and Figure 3). Although the hypothetical complex of SLP2 and other mitochondrial proteins is likely one of numerous incompletely characterized mitochondrial complexes, many of them might behave analogically in disordering conditions. Thus, immunolabeling of SLP2 released from its complex may serve a unique role as a marker of general mitochondrial dysfunction and cellular disorder.

## 5. Using Type 2 Mitochondria for Identification of Necrosis-like Cellular Ultrastructural Pathology in Mouse Brain

**Figure 4:**
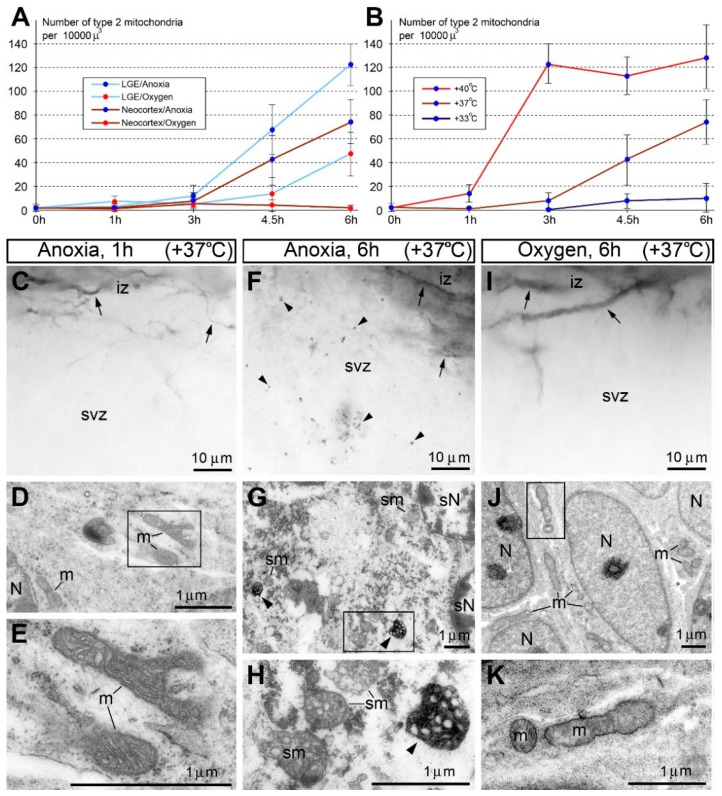
Anoxia increases, whereas oxygenation attenuates, the rise of immunopositive type 2 mitochondria and necrosis-like ultrastructural pathology in mouse embryo brains. (**A**) Quantification of immunopositive mitochondria in the subventricular zone (svz) of neocortex or lateral ganglionic eminence (LGE) in anoxic conditions or with oxygenation. (**B**) Growth of the density of type 2 mitochondria after anoxia is increased at high temperature (40 °C) and decreased at low temperature (33 °C) compared with physiological temperature (37 °C). In (**A**,**B**), the Y-axis shows the average from 6 embryos ± SD. (**C**–**K**) Representative light (**C**,**F**,**I**) and electron micrographs (**D**,**E**,**G**,**H**,**J**,**K**) of anti-CB_1_R-L31 labeling of the neocortical svz and intermediate zone (iz) under the indicated conditions. Normal CB_1_R-positive axons are visible in iz (arrows in **C**,**F**,**I**). (**C**–**E**) At 1 h of anoxia treatment, neurons retain normal structure of nuclei (N) and organelles, including mitochondria (m) that are negative for anti-CB_1_R-L31 labeling. (**F**–**H**) At 6 h of anoxia, neurons show numerous immunopositive mitochondria (arrowheads) and necrosis-like ultrastructural pathologies, such as spherical nuclei (sN) and swollen mitochondria (sm). (**I**–**K**) In contrast, embryo brains immersed in the oxygenated medium demonstrate very few, if any, immunopositive mitochondria (**I**) and mostly normal ultrastructure of neurons (**J**,**K**). Framed areas in **D**,**G**,**J** are enlarged in **E**,**H**,**K**, respectively. Modified from [16]. (License 6183801444721).

Immunohistochemical labeling of SLP2 with anti-CB_1_R-L31 serum represents a convenient method for identification of disordered cells because the type 2 mitochondria are well detectable with light microscopy and can be a subject of quantitative analysis for characterizing cellular functionality in certain experimental conditions. For example, we sporadically observed the type 2 mitochondria in developing neurons and endothelial cells of generally healthy mouse embryo brains [6]. We also detected that emergence of the type 2 mitochondria in certain cells correlated with the necrosis-like ultrastructural pathology of these cells, while adjacent cells containing immunonegative mitochondria showed normal ultrastructure. Anoxic conditions applied to mouse embryo brains, as well as hypoxia–ischemia of adult mice provoked a marked increase in the type 2 mitochondria in the neuropil in parallel with massive necrosis-like cell death [16]. In particular, we analyzed CB_1_R-L31 immunolabeling in isolated mouse embryo brains that were exposed to anoxic conditions through immersion in a saline solution. As a control, we used isolated mouse embryo brains immersed in the saline solution bubbled with oxygen. In anoxic conditions, a marked increase in the type 2 immunopositive mitochondria in all brain segments was observed after 4.5 and 6 h of incubation, whereas their numbers were reduced in the oxygenated group (Figure 4A). The low number of immunopositive mitochondria and generally normal cellular ultrastructure in ≤3 h of anoxia may reflect mammalian embryos’ high resistance to hypoxia [34,35,36]. We also detected that the numbers of type 2 mitochondria are increased at high temperatures (40 °C) and decreased at low temperature (33 °C) in comparison with the physiological temperature (37 °C) during anoxia (Figure 4B). Consistently, emergence of the type 2 mitochondria was unequivocally linked to the necrosis-like ultrastructural pathology of neurons (Figure 4C–K). Importantly, ultrastructural evidence of other cell death mechanisms, such as apoptosis or autophagy, was not encountered in the embryo brains exposed to anoxia. This indicates that the anoxia-induced necrosis-like cell death mechanism is virtually undetectable using biochemical or immunochemical methods such as terminal deoxynucleotidyl transferase dUTP nick end labeling (TUNEL) assay, caspase immunolabeling, or labeling of autophagy markers. Our findings also provide evidence that the mechanism of hypothermia therapy of neonatal hypoxic–ischemic encephalopathy [37,38,39,40] is based on suppression of necrosis rather than apoptosis. Apoptosis results in controlled cell shrinkage and fragmentation via caspase activity, as well as an anti-inflammatory cytokine release. In contrast, necrosis signals (e.g., RIP1 and RIPK1) lead to cell swelling, lysis, and a pro-inflammatory cytokine release that diffuses through the surrounding tissue [41,42]. Necrosis represents the most destructive cell-death pathway, whereas apoptosis locally eliminates damaged cells and may serve to protect the tissue and improve the survival of the organism. Thus, immunolabeling of the type 2 mitochondria serves as a target for detecting derangement of mitochondria and necrosis-like cell death in situ. Anti-CB_1_R-L31 serum provides a unique opportunity for selective visualization of disordered mitochondria. We suggest using this serum because none of the currently available SLP2 antibodies provide immunohistochemical labeling of mitochondria.

## 6. Disorder of Golgi Apparatus Precedes Anoxia-Induced Pathology of Mitochondria

**Figure 5:**
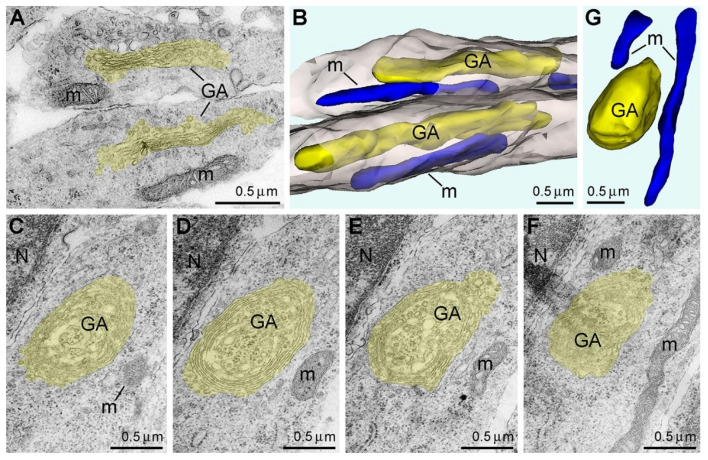
Electron micrographs and 3D reconstructions of Golgi apparatus (GA) and mitochondria from mouse embryo cerebrum fixed immediately after decapitation (**A**,**B**) and after 1 h of anoxia (**C**–**G**). GA including cisternae and vesicles are shown as yellow in the electron micrographs (**A**,**C**–**F**) and the 3D images (**B**,**G**). Mitochondria (m) are depicted as blue in the 3D images. Two adjacent neuronal processes are displayed as semi- transparent grey in the 3D image (**B**). In the control brain, both analyzed GA show normal stacks of cisternae and elongated configuration. Serial micrographs (**C**–**F**) and 3D reconstruction (**G**) demonstrate spherical onion-like shape of GA after 1 h of anoxia. Abbreviation: N, cell nucleus. Modified from [33]. (License CC BY).

We applied electron microscopy with 3D reconstruction to quantitatively analyze the mitochondria and other organelles during the first hours of anoxia. Although our quantitative study showed an approximately 3 h long delay for type 2 mitochondria upregulation (Figure 4A), we detected reshaping of the Golgi apparatus (GA) after only 1 h of anoxia. Thus, GA disorder is the earliest morpho-functional reaction of mouse embryo brains to anoxic conditions. In particular, GA showed concentrical swirling of the cisternae forming a spheroid with the trans-cisterna in the center of the sphere that contrasted with the usual stack of cisternae in normally functioning GA (Figure 5 and [33]). Similar reshaping of GA was described in mammalian cells at several pathological conditions and named onion-like GA [43,44]. Such disturbance of GA likely interferes with its function for post-translational protein modification and secretory trafficking [45]. It is characteristic that the onion-like GA phenotype was observed before other organelles, including mitochondria, showed ultrastructural disorder. Analyses after 3 h of anoxia confirmed swirling in almost 100% of GA and swelling in the majority of mitochondria. At this time point, the length of the mitochondria was significantly decreased, and the diameter was increased so the mitochondria took on a nearly spherical shape [33].

**Figure 6:**
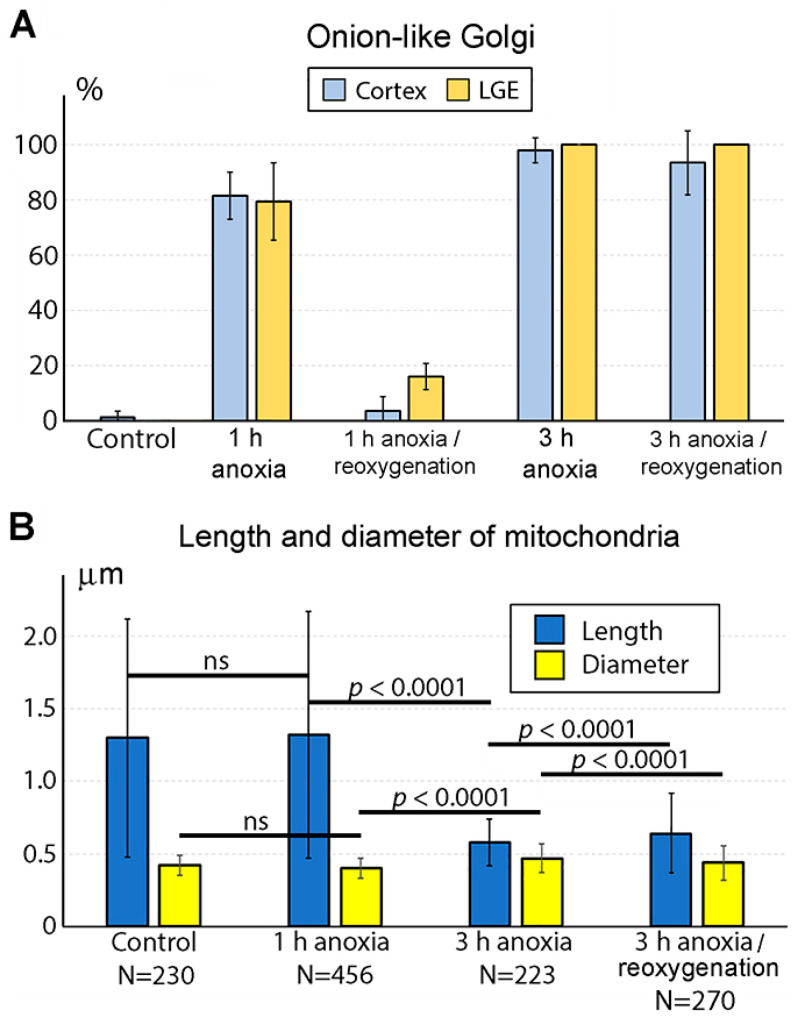
(**A**) Upregulation of swirled onion-like GA in the anoxia-exposed mouse embryo cerebral cortex and lateral ganglionic eminence (LGE). Swirled GA are nearly absent in control animals. Normal GA are virtually absent after 3 h of anoxia. Reoxygenation of the anoxia-exposed mouse embryo brains decreases the percentage of the onion-like GA after 1 h but not after 3 h of anoxia. (**B**) Morphometric characterization of mitochondria in mouse embryo cerebrum after indicated time of anoxia or reoxygenation. Notice the large difference between 3 h and 1 h anoxia groups. Reoxygenation after 3 h of anoxia produces generally minor (although statistically significant) effects on the mitochondria. The Y axis shows mean ± SD for each group. Ns are numbers of analyzed 3D-reconstructed mitochondria. “ns” indicates absence of statistically significant difference. Modified from [33]. (License CC BY).

We also found that the reaction of GA to anoxia may be reversible in the case of fast reoxygenation. After 1 h of anoxia with subsequent return of the embryo brains to the oxygenated liquid, we observed a recovery of normal ultrastructure of most GA. Namely, in the cerebral cortex, only 3 GA among a total of 71 identified showed the onion-like phenotype, and only 8 out of 50 GA were swirled in the lateral ganglionic eminence (Figure 6A). Other organelles, including mitochondria, also demonstrated normal ultrastructure, although we did not perform 3D and morphometric analysis after 1 h anoxia/reoxygenation. In contrast, reoxygenation after 3 h of anoxia does not reverse the damage of the cells. In particular, among 29 GA identified in the cerebral cortex, 27 showed the onion-like phenotype. Analogically, in the lateral ganglionic eminence, no normal GA were identified, while 29 GA showed the onion-like phenotype (Figure 6A). A morphometric analysis of mitochondria in the cerebral cortex after 3 h of anoxia with subsequent reoxygenation showed a minor, although statistically significant, increase in the average length and a decrease in the average diameter of the mitochondria in comparison with the 3 h anoxia group. At the same time, the ranges of the length and the diameter (measured as the standard deviations of the means) in the reoxygenation group increased (Figure 6B). This indicates heterogeneity in mitochondrial reactions. While some mitochondria return to healthy morpho-functional characteristics, others may be disrupted further. As a result, we conclude that a majority of GA can reestablish morpho-functional characteristics if the oxygen supply returns after 1 h of anoxia when the mitochondria show a normal ultrastructure. Reoxygenation does not allow the GA ultrastructure to recover after 3 h of anoxia when the mitochondrial disruption is significant and irreversible.

According to the observed normal ultrastructure of mitochondria after 1 h of acute anoxia, oxygen deficit is likely to reduce oxidative phosphorylation and ATP production without a morphologically detectable disruption in the energy-production machinery. Nevertheless, a lack of ATP apparently provokes the observed ultrastructural pathology and dysfunction of the GA. Such dysfunction may be reversible if the oxygen supply returns soon. In case the energy deficit continues long enough, the GA malfunction may damage other organelles, including mitochondria. In turn, damaged mitochondria may initiate numerous irreversible reactions, such as reactive oxygen species production, calcium leakage, cytochrome C leakage, cell apoptosis or necrosis, etc. [23,46,47]. Thus, the molecular architecture of mitochondria, at least in mouse embryo brains, is resistant to anoxia for approximately 1 h, whereas intracellular transportation and other functions executed by the GA are vulnerable to a shortage of ATP supply and likely participate in subsequent cellular disruption, including massive fission and swelling of mitochondria that were detected after 3 h of anoxia. During the next step of disorder, after 4.5 h of anoxia, we detected the exponential upregulation of the type 2 mitochondria, which likely indicates dissociation of mitochondrial protein complexes, making the mitochondria irreversibly unfunctional. Thus, a dramatic increase in the quantity of type 2 mitochondria likely signifies the point of no return for the necrosis-like death of cells in the mouse brain.

## 7. Type 2 Mitochondria Identify Necrosis-like Cell Death in the Liver and White Adipose Tissue

**Figure 7:**
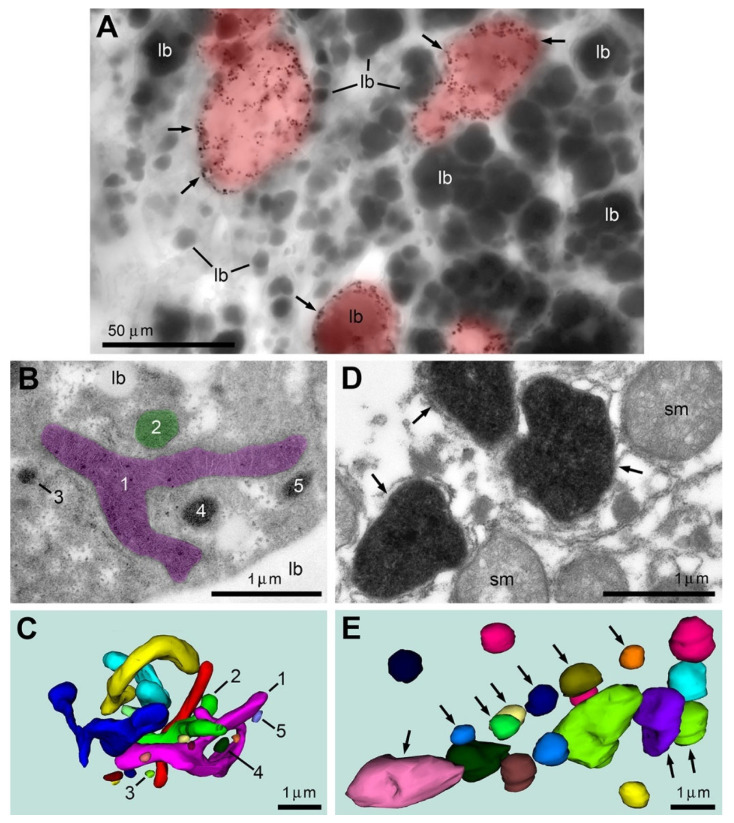
Immunolabeling using CB_1_R-L31 serum identifies SLP2-like type 2 immunopositive mitochondria and reveals necrosis-like ultrastructural pathology among hepatocytes from aging mice. (**A**) Representative light micrograph shows numerous type 2 mitochondria (arrows) in a fraction of hepatocytes (highlighted red) in the liver of a 16-month-old mouse. Numerous big lipid bodies (lb) are characteristic for hepatocytes. (**B**,**C**) Hepatocytes containing only immunonegative mitochondria demonstrate generally normal ultrastructure of organelles and high variability of mitochondrial shape although all mitochondria show well-developed crista and dense matrix. For example, mitochondria #1 and #2, correspondingly colored magenta and green in the electron micrograph (**B**) and 3D reconstruction from serial sections (**C**), show elongated and branching shapes. Mitochondria #3–5 are small and spherical. (**D**,**E**) Type 2 mitochondria-containing cells from the same sample reveal fission and swelling of all mitochondria. Other organelles are hardly identifiable in the type 2 mitochondria-containing cells demonstrating their necrosis-like ultrastructural pathology. Type 2 mitochondria are indicated with arrows in the electron micrograph (**D**) and the 3D reconstruction (**E**); immunonegative swollen mitochondria (sm) are not pointed in the 3D image. Distinct mitochondria are depicted in different colors in the 3D images (**C**,**E**).

We investigated if CB_1_R-L31 serum can be used for identification of disordered mitochondria in other tissues and organs. We chose mouse liver and white adipocytes because these cells are morpho-functionally distinct from brain cells. In the liver from postnatal mice, we very rarely observed SLP2-like type 2 immunopositive mitochondria in five analyzed young (11-day-old) mice, whereas they were numerous in two analyzed aged (16-month-old) mice. Our correlative light/electron microscopy investigation confirmed that immunopositive dots visible in light microscopy represent accumulations of DAB-Ni immunoperoxidase reaction end product in the mitochondrial matrix (Figure 7) as we observed in the brain [6,16]. It is characteristic that immunopositive mitochondria concentrate in distinct hepatocytes, while they are absent from adjacent cells. In the liver of the aged mice, like in the developing brain, the type 2 mitochondria are linked to mitochondrial fission and swelling paralleled with destruction of other organelles showing necrosis-like cellular ultrastructural pathology. Adjacent immunonegative cells contain long-branched mitochondria and preserve the normal ultrastructure of functional hepatocytes (Figure 7).

**Figure 8:**
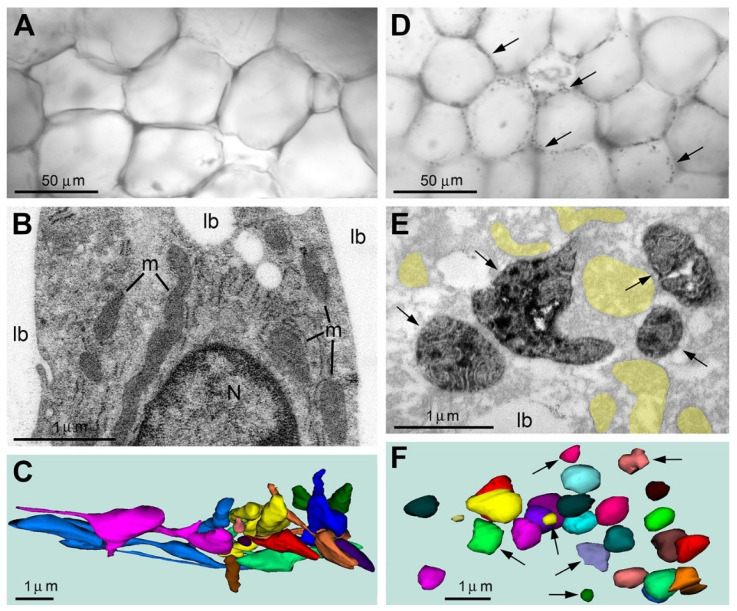
Type 2 SLP2-like immunopositive mitochondria indicate necrosis-like ultrastructural pathology in a fraction of white adipocytes from young adult mouse abdomen. Immunolabeling using CB_1_R-L31 serum showed a negative reaction in a majority of adipocytes (**A**–**C**), whereas immunopositive mitochondria were detected in certain tissue segments (**D**–**F**). (**A**) Representative light micrograph of typical immunonegative adipocytes. (**B**) Electron micrograph of a segment of white adipocyte with the nucleus (N) and cytoplasm sandwiched between two big lipid bodies (lb). Notice the immunonegative mitochondria (m) with dense matrix. (**C**) 3D reconstruction of mitochondria (shown in distinct colors) from serial sections of an immunonegative adipocyte. Most mitochondria show elongated and branching shapes. Evidence of swelling of the matrix was not encountered, while enlarged segments of the mitochondria in (**C**) contain electron-dense matrix characteristic of functional mitochondria. (**D**–**F**) An arbitrarily chosen fragment of white adipose tissue contains numerous type 2 immunopositive mitochondria (arrows) as visible under light (**D**) and electron microscope (**E**). Immunonegative remnants of mitochondria are highlighted yellow. The immunoperoxidase reaction DAB-Ni end product is located in the matrix of the mitochondria while cristae are immunonegative. 3D reconstruction of mitochondria (shown in distinct colors) from serial sections of an adipocyte identifies that the type 2 mitochondria (arrows) as well as immunonegative ones (not pointed) are swollen and spherical-shaped.

Using anti-CB_1_R-L31 serum and correlative light/electron microscopy, we also detected SLP2-like immunopositive type 2 mitochondria in the abdominal white adipose tissue from adult mice (N = 6). Although the main body of the adipose tissue remains immunonegative, we detected immunopositive granules in the cellular periphery of many adipocytes. Electron microscopy confirmed that a DAB-Ni immunoreaction end product was accumulated in the matrix of spherically shaped swollen mitochondria (Figure 8). Like in the brain and liver, emergence of the type 2 mitochondria in the adipocytes was accompanied by fission and swelling of all the mitochondria and disorder of other organelles and cytoplasm. Thus, immunohistochemical labeling using CB_1_R-L31 serum identifies a necrosis-like ultrastructural pathology of white adipocytes. At the same time, the immunonegative compartments of the adipose tissue preserve normal organelles and long-branching mitochondria characteristic of functional adipocytes. Distribution of the immunopositive adipocytes, their relative number and the reason for their appearance are currently unclear. Overall, our application of CB_1_R-L31 serum for brain, liver and adipose tissue indicates that immunohistochemical detection of SLP2-like immunopositive type 2 mitochondria can serve as a marker for identification of a necrosis-like ultrastructural pathology and cell death in different tissues and organs.

## 8. Dissociation of Mitochondrial Protein Complexes Identifies Necrosis-like Cell Death

**Figure 9:**
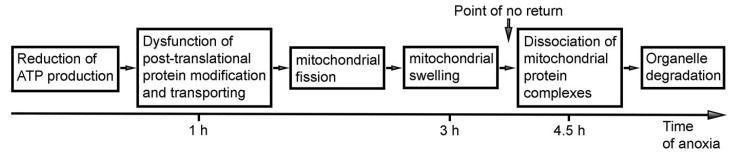
Hypothetical model of the consequence of cellular events during anoxia in mouse embryo brain. Reduction of ATP production due to lack of oxygen (or other substrates) is reversible if the molecular machinery stays intact until the supply returns. Our data show that disorder of GA—a critical component of the protein modification and transportation system—may be temporary if oxygen supply returns. Reciprocally opposite processes of mitochondrial fission and fusion are known to be balanced in functional cells [16,25,33,48,49,50,51,52]. Massive mitochondrial swelling is a good candidate for the point of no return because ATP synthesis depends on H^+^ concentration in the intermembrane space. Nevertheless, we do not know if a mitochondrion can remove the excess of water and restore the functionality if most proteins and complexes are intact. Dissociation of the mitochondrial protein complexes in the majority of mitochondria unequivocally stops ATP production and prevents cellular recovery.

Identification of disordered mitochondria in different organs and cell types suggests the universal character of SLP2 immunolabeling and wide applicability of anti-CB_1_R-L31 serum for identification of malfunctional mitochondria and the disordered cells that contain them. Because many mitochondrial protein complexes likely behave similarly in pathological conditions, SLP2 released from the complex with other mitochondrial proteins may serve as a novel immunohistochemical marker of mitochondrial dysfunction and cellular disorder. The mitochondrial and cellular disorders identified here occur sporadically in normal brains and other organs and ubiquitously in hypoxia-exposed developing and adult mouse brains. We propose using anti-CB_1_R-L31 serum for in situ identification of dissociating mitochondrial protein complexes which likely indicate a point of no return during necrosis-like cell death (Figure 9). Although we do not know the epitope of antibody binding, our findings open up the possibility of using anti-CB_1_R-L31 serum as a tool for immunohistochemical exploration of the role of SLP2 in mitochondria under normal and pathological conditions. In our hands, the method was effective for different types of cells and tissues, such as central nervous and vascular systems, hepatocytes and white adipocytes. The observed mitochondrial phenotype provides a new target for diagnosis of necrosis-like cell degeneration in different organs. Our original experimental approach partially covers the gap in the contemporary methodical arsenal complementing popular detection of nuclear and cytoplasmic reactions (e.g., TUNEL assay and active caspase immunolabeling) that reveal the apoptosis-like regulated cell death.

## 9. Piercing Nuclear Hernia Is a Novel Ultrastructural Pathology of Migrating Cells

**Figure 10:**
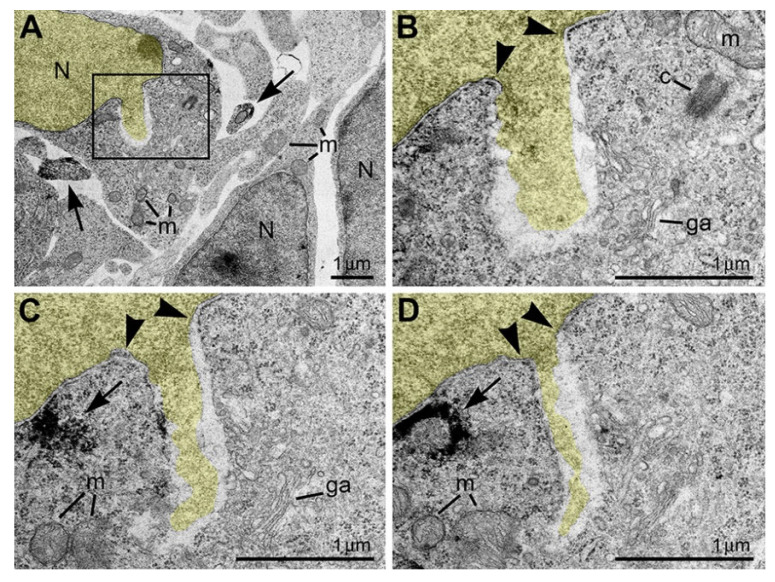
NE rupture in a neuron from the cortical plate of wild-type mouse embryo exposed to CB_1_R agonist CP-55940. (**A**) Low-power micrograph shows ultrastructure of neuronal cell bodies and neuropil that is generally normal for the developmental stage. (**B**–**D**) Serial high-power images of NE rupture from the framed area in (**A**). Chromatin stream expelled from the nucleus (highlighted yellow) is not surrounded by the nuclear membrane. A complete series of 12 ultrathin sections show that the chromatin stream stays in cytoplasm. Arrowheads indicate points of interruption of the nuclear membrane. Arrows point to CB_1_R-positive axons in (**A**) and CB_1_R-positive intracellular vesicle in (**C**,**D**). Abbreviations: c, centriole; ga, Golgi apparatus; m, mitochondria; N, nucleus. Modified from [53]. (License CC BY).

**Figure 11:**
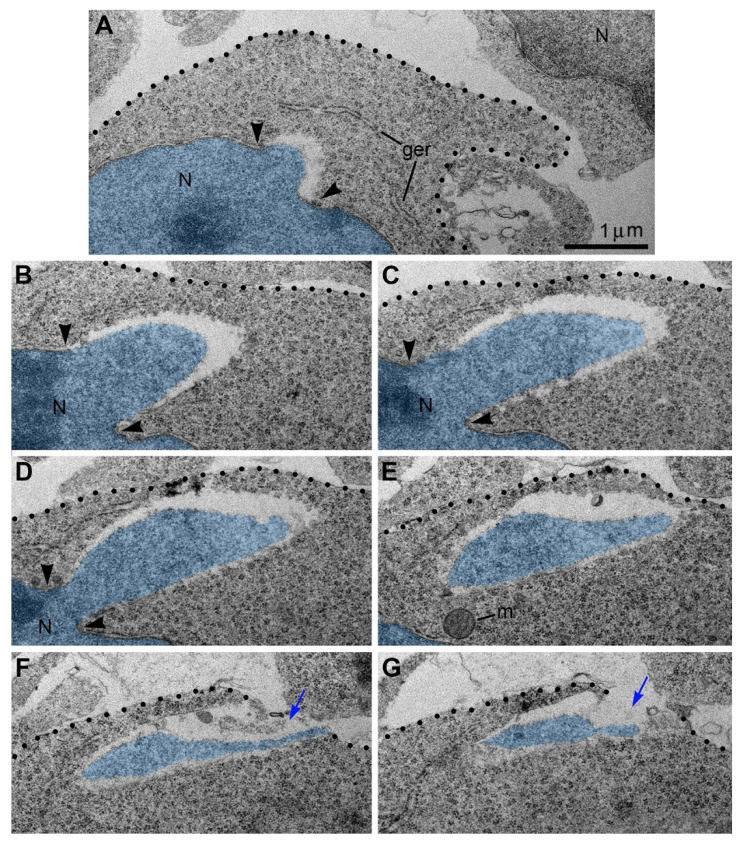
Serial micrographs of PNH in a neuron from the cerebral intermediate zone of an embryo exposed to CB_1_R agonist WIN-55,212-2 show continuum of the chromatin stream expelled from the nucleus (highlighted blue) and penetrating the intercellular space. Points of NE interruption in (**A**–**D**) are indicated with arrowheads. Volume of the herniated chromatin stream was estimated in the complete series of 31 sections as 1.63 µm^3^. Blue arrows in (**F**,**G**) denote the interruption of the plasma membranes, which are designated with dotted lines. Scale bar in (**A**) is valid for all. Abbreviations: ger, granular endoplasmic reticulum; m, mitochondrion; N, nucleus. Modified from [53]. (License CC BY).

Recently, we applied electron microscopy of ultrathin sections for complete 3D reconstructions of nuclei and cell bodies for analysis of neurons from CB_1_R^−/−^ and wild-type mouse embryos exposed to CB_1_R agonists. We documented local breakups of the inner and outer nuclear membranes accompanied by herniation of chromatin into the cytoplasm (Figure 10 and [53]). Similar cellular disorders named nuclear envelope (NE) ruptures were previously identified in developing neurons and metastatic cancer cells [54,55,56,57,58]. Surprisingly, in a fraction of migrating neurons, streams of herniated chromatin ruptured not only the NE but also the plasma membrane, exposing cytoplasm and nucleoplasm to the intercellular space (Figure 11). As it was the first description of a simultaneous rupture of the NE and the plasma membrane, we named this cellular pathology “piercing nuclear hernia” (PNH).

**Figure 12:**
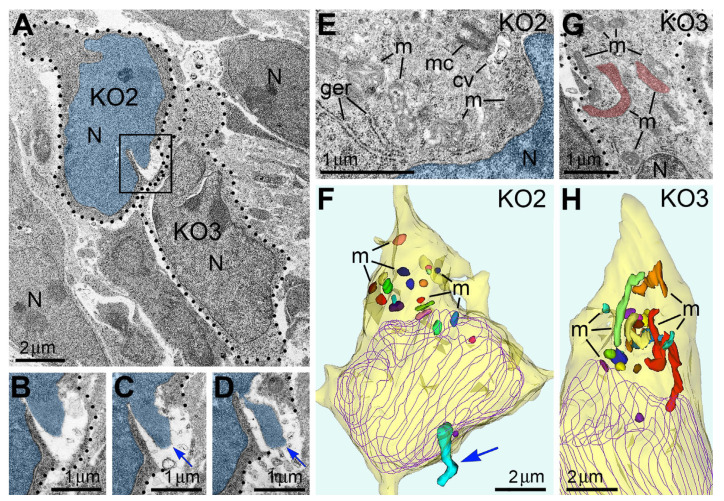
Adjacent herniated and non-herniated neurons show distinct ultrastructural characteristics of mitochondria. (**A**) Two neurons from the intermediate zone of CB_1_R^−/−^ embryo indicated as cell numbers KO2 and KO3 (dotted lines designate their plasma membranes). Cell KO2 exhibits PNH, whereas KO3 shows an intact nucleus. (**B**–**D**) Serial images of PNH from the framed area in (**A**). The chromatin stream (blue arrows) herniated from the nucleus (highlighted blue) penetrates the intercellular space. (**E**–**H**) High-power images (**E**,**G**) and 3D reconstructions (**F**,**H**) of the cells KO2 and KO3. Twenty randomly selected mitochondria from each cell are shown in different colors in the 3D reconstructions. Cell KO2 exposing PNH (blue arrow) contains mostly short or spherical mitochondria, whereas several mitochondria from cell KO3 are long. Nuclei profiles are traced violet. Abbreviations: cv, cilial vesicle; ger, granular endoplasmic reticulum; m, mitochondria, mc, mother centriole; N, nucleus. Modified from [53]. (License CC BY).

Rupture of the nuclear and plasma membranes in cases of PNH severely disrupts the barrier function of the membranes and obviously results in fast death of the cell that should be qualified as regulated cell death (RCD; see recommendations of the Nomenclature Committee on Cell Death [59]). Belonging to a divergent group of RCD mechanisms is defined as a consequence of the fall of adaptive responses to environmental perturbations [59]. Numerous mechanisms of RCD can be crudely classified into three complex groups: apoptosis-, autophagy-, and necrosis-type cell-death mechanisms [41,42,59,60,61,62,63,64,65]. In contrast to RCD, cells exposed to extreme physical, chemical, or mechanical stimuli may immediately lose their structural integrity and die in an uncontrollable manner termed “accidental cell death” [59]. Cases of accidental cell death are virtually undetectable using biochemical or immunochemical methods suitable for RCD, such as TUNEL assay, active caspase immunolabeling, or labeling of autophagy markers. Electron microscopy, on the other hand, visualizes organelles regardless of their functional conditions and can detect dead cells or cellular remnants. Accordingly, we found that the emergence of PNH in migrating neurons is often paralleled with an ultrastructural pathology of the organelles. In particular, cells with PNH demonstrated short mitochondria, while adjacent non-herniated cells showed normally sized mitochondria (Figure 12). A reduction in mitochondrial length—predominance of fission over fusion—may serve as evidence of disordered cellular energetics or other malfunctions [23,25,26,48,49,50,51,66]. Involvement of disordered endocannabinoid signaling in the PNH emergence (see below) indicates belonging to RCD mechanisms. On the other hand, the abrupt loss of the barrier function of the nuclear and plasma membranes may result in fast death of the cell, i.e., suggests the executive mechanism of cell death that resembles the accidental cell death. Thus, PNH represents a novel type of cell pathology that is likely lethal for the cell. After extensive study, the procedure of PNH upregulation may be useful for inducing breaks of the plasma membrane and death of hazardous cells, for example, migrating metastatic tumor cells (see [67] for discussion).

## 10. Cannabinoid Signaling Plays a Role in Cytoskeleton Functionality

**Figure 13:**
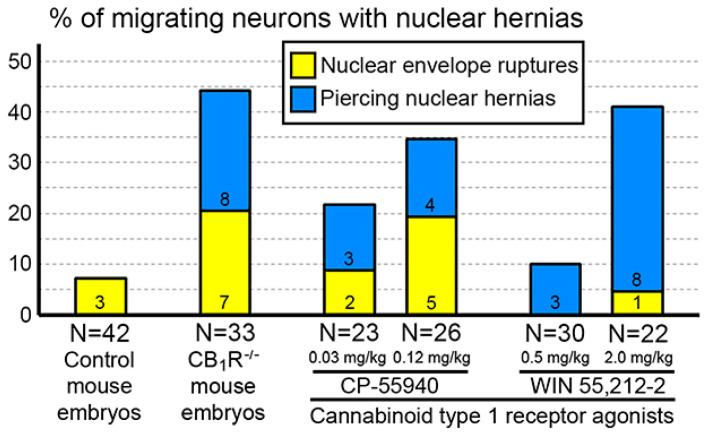
Percentages of cells with NE ruptures and PNHs in the embryo cerebral cortex of wild-type mice, CB_1_R^−/−^ mice and wild type mice exposed to CB_1_R agonists. Numbers of cells with NE ruptures and PNHs are indicated in each column. Ns indicate the numbers of analyzed cells from each group. Modified from [53,67]. (License CC BY).

We showed that the probability of chromatin herniation is increased in cases of disorder of the endocannabinoid system [53]. Indeed, numerous migrating neurons in CB_1_R^−/−^ mouse embryos and wild-type embryos exposed to different agonists of CB_1_R show NE ruptures or PNHs (Figure 13). This indicates that deviations from optimal functioning of the endocannabinoid system in under- or over-activity may trigger mechanisms increasing the membrane’s vulnerability and chromatin herniation.

The large volume of herniated chromatin [53] indicates that NE ruptures and PNHs in nuclei moving through the developing cerebrum are consequences of extremely high intranuclear pressure. In accord, the commonly accepted mechanism of NE rupture includes increased intranuclear pressure and cytoskeleton malfunction [54,56,68,69,70,71,72,73,74,75]. A link between the endocannabinoid system disorder and NE ruptures and PNHs provides evidence that optimal endocannabinoid signaling is crucial for cytoskeleton functionality in migrating neurons. Maintenance of the shape of mature neurons, as well as, their reorganization during migration are determined by numerous cytoskeleton components, such as microtubules, neurofilaments, actin, myosin II, centrosomes, and others [76,77,78,79,80,81,82,83,84,85,86]. We recently reviewed the possible role of the endocannabinoid system in actomyosin cytoskeleton functionality in developing and mature neurons from different species [53,67]. Overall, deviated expressions of the cytoskeletal components after stimulation or knock-out of CB_1_R establish evidence of cannabinoid signaling participation in neuronal cytoskeleton consolidation. Our discovery of PNH in migrating neurons gives us an opportunity to study enigmatic molecular mechanisms of the endocannabinoids’ action in the developing brain.

## 11. Role of the Cytoskeleton in Lateral Expansion of the Cerebral Cortex

**Figure 14:**
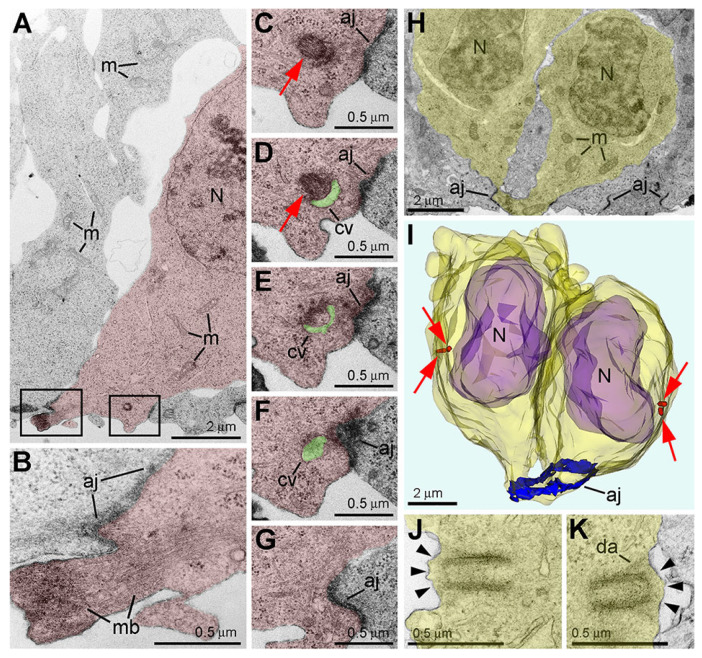
Electron microscopy analysis of the centrosome anchorage in apical NPCs in cerebral ventricular zone from 9-day-old (**A**–**G**) and 16-day-old (**H**–**K**) mouse embryos. (**A**) Low-power electron micrograph of a dividing cell (highlighted red) containing microtubule midbody (mb) in the apical segment characteristic for late cytokinesis (left frame in **A** enlarged in **B**). The right framed area was enlarged in serial micrographs (**C**–**G**) showing the centriole attached to the cilial vesicle (cv, highlighted green) without cilium. Centrosome (red arrow) is located adjacent to adherens junction (aj) and the apical membrane, but it is not anchored to the membrane in the moment of fixation. (**H**) Low-power electron micrograph of a serial section of a dividing cell (highlighted yellow). (**I**) 3D reconstruction of prospective daughter cells (semi-transparent yellow) that are still interconnected in the apical segment. Centrioles are depicted in red and pointed to with red arrows; the daughter nuclei (N) are semi-transparent violet, and the adherens junction belt (aj) is blue. (**J**,**K**) Two centrioles from both daughter cells are anchored to the baso-lateral membrane (arrowheads) by distal appendages (da). Abbreviation: m, mitochondria. Modified from [94]. (License 6183791501210).

**Figure 15:**
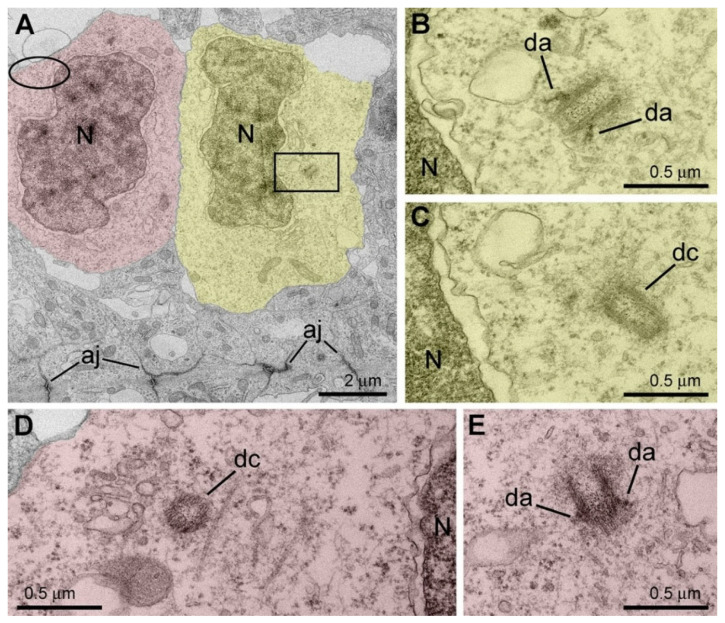
Electron microscopy analysis of an apical NPC in the cerebral ventricular zone from a 54-day-old rhesus macaque embryo. (**A**) Low-power micrograph shows two daughter cells shortly after mitosis (highlighted yellow and red). The rectangularly framed area is enlarged in serial sections (**B**,**C**) depicting both centrioles located free in cytoplasm. Ovoid in (**A**) indicates approximate position of centrosome in the other daughter cell shown in high-power serial not adjacent sections in (**D**,**E**). Both centrioles are free in cytoplasm. Distal appendages (da) identify mature mother centrioles. Abbreviations: aj, adherens junctions; dc, daughter centriole; da, distal appendage; N, nucleus. Modified from [94]. (License 6183791501210).

**Figure 16:**
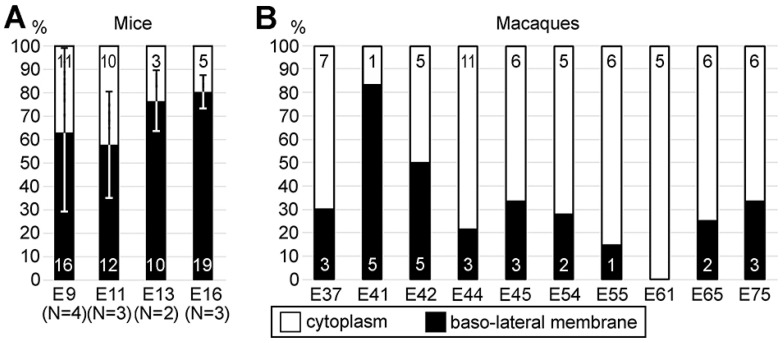
Quantifications of anchored versus free centrosomes in the cells shortly after mitosis from cerebral ventricular zone of mice (**A**) and rhesus macaques (**B**) at different embryonic days (E9-E75). Numbers of identified centrosomes are indicated in corresponding columns. The quantifications in mouse embryos (**A**) are represented as mean ± SD. N indicates the number of analyzed mouse embryos of certain age. Notice higher numbers of anchored centrosomes in mice of all ages and generally lower numbers in macaques. Modified from [94]. (License 6183791501210).

Initiating study of mitotic cells in the cerebral ventricular zone, we expected to find a role of cannabinoid signaling in neurons’ proliferation. However, during the investigation, we found no evidence of the cannabinoid receptors’ expression in dividing cells in mouse and rhesus macaque cerebral cortices [87]. Instead, we discovered another aspect of cytoskeleton function. We used the recognizable ultrastructure of centrosomes, which play a key role as a microtubule organizing center [82,84,86], for quantitative morpho-functional characterization of the cytoskeleton. We identified significant species-specific differences in the proportion of anchored versus free centrosomes in dividing apical neural progenitor cells (NPCs). Anchorage of the mother centriole to the apical membrane of NPCs is a fundamental part of repetitive circles of cell divisions in the apical region of the developing telencephalon [82,84,86,88,89,90]. In contrast, baso-lateral anchorage of the mother centriole predisposes the cell for delamination from the apical surface and its basal migration, after which the cell may continue proliferation as a basal NPC but does not participate in lateral expansion of the prospective cerebral cortex [91,92,93]. In particular, we analyzed positions of centrosomes in the apical NPCs shortly after mitosis [94]. We showed that mother centrioles may be anchored to the plasma membrane basally relative to the adherens junction belt (not to the apical segment of the plasma membrane) or stay free in cytoplasm (Figure 14 and Figure 15). Free post-mitotic centrosomes preserve the chance to anchor either baso-laterally or apically [95]. The apical position (below the adherens junction belt) provides a faster link between the daughter cell and the ventricular surface, thus increasing the probability of locating the cell at the ventricular surface as an apical NPC during the next cell cycle [91,96,97,98,99]. Quantification of anchored and free centrosomes showed characteristic differences between the species. In mice, 66.3% of centrosomes were anchored (namely, we identified 86 anchored and 57 free centrosomes), whereas in monkeys, only 31.8% centrosomes were anchored to the baso-lateral membrane (27 anchored centrosomes and 58 free ones) [94]. In mice, anchored centrosomes were more numerous than free ones in all embryonic age groups studied. The ratio of anchored versus free cytoplasmic centrosomes was much lower in the macaque embryos (Figure 16). The dynamics of centrosome anchorage might represent a mechanism that impacts transformation of apical NPCs into basal NPCs. In particular, the increased apical anchorage of NPCs may prolong apical neurogenesis and lateral expansion of the cerebral cortex. Thus, our findings suggest a role of centrosomes in the maintenance of ventricular neurogenesis that may increase the size of the cerebral cortex in rhesus macaques and other gyrencephalic animals. Our observations afford evidence strengthening the radial unit hypothesis of cerebral expansion [100,101,102,103,104,105,106,107]. The hypothesis proposes that an increased rate of ventricular proliferation produces a larger number of radial units, resulting in an expanded cellular sheet in the cerebrum, which later buckles and transforms a lissencephalic cerebral cortex into a gyrencephalic one. This is an extremely important feature because the size of the cerebral cortex is the basis of high cognitive abilities, culminating in humans’ capacity for thinking and language [108,109,110,111,112,113,114,115,116].

## 12. Conclusions

In this article, we reviewed selected studies from our laboratory which highlight unique opportunities presented by electron microscopy of ultrathin sections.

In particular, our comprehensive study of CB_1_R immunolabeling led to the discovery of double selectivity of anti-CB_1_R-L31 serum, which in addition to CB_1_R, also recognized the mitochondrial protein SLP2. This finding turned out to be instrumental for in situ identification of mitochondrial protein complexes’ dissociation in cells undergoing necrosis-like death. Such dying cells are undetectable using currently available methods for identification of apoptosis and autophagy. Application of this novel method combined with transmission electron microscopy let us analyze the time course of the morphological disorder of organelles at pathological conditions. For example, we observed that Golgi apparatus cisternae swirling preceded anoxia-induced disorder of other organelles, including mitochondria, in mouse embryo brains. Such a Golgi apparatus disorder was reversible in the case of quick reoxygenation.

Using electron microscopy of serial ultrathin sections enabled our unequivocal identification of a new type of ultrastructural cell pathology. Namely, we identified chromatin streams that herniated from the nucleus and ruptured through both the nuclear envelope and the plasma membrane. We named this cellular pathology “piercing nuclear hernia”. Moreover, we showed that abnormalities of endocannabinoid signaling upregulated ruptures of the nuclear and plasma membranes in migrating cerebral neurons. This grants a new look at as-of-yet unknown mechanisms of marijuana’s effects on the human fetus brain.

High resolution of transmission electron microscopy enabled us to identify the characteristic locations of extremely small organelles such as centrosomes, which serve as the main cytoskeleton organizing center during and after cellular division. We found that lateral expansion of the developing cerebral cortex correlates with a species-specific ratio of free versus anchored centrosomes in the apical neural progenitor cells shortly after mitosis. In mice, centrosomes showed a high rate of anchorage to the plasma membrane. In rhesus macaques, in contrast, prolonged free cytoplasmic location of the centrosomes provides more opportunity for lateral expansion of the cerebral cortex—the critical basis of primates’ cognitive abilities.

Despite the development of new morphological methods and improved access to equipment for super-resolution fluorescent microscopy and volume electron microscopy, transmission electron microscopy of ultrathin sections provides opportunities for multiple unique experimental approaches. We hope that the above examples of successful studies will encourage young and experienced scientists to utilize classical methods of transmission electron microscopy.

## Data Availability

No new data were created or analyzed in this study. Data sharing is not applicable to this article.
